# Neck emergency due to parathyroid adenoma bleeding: a case report

**DOI:** 10.1186/1752-1947-3-7404

**Published:** 2009-05-21

**Authors:** Isabella Merante-Boschin, Matteo Fassan, Maria Rosa Pelizzo, Eric Casal Ide, Massimo Rugge

**Affiliations:** 1 Department of Medical & Surgical Sciences, Special Surgical Pathology Unit, University of Padova, Padova, Italy; 2 Department of Medical Diagnostic Sciences & Special Therapies, Pathology Unit, University of Padova, Padova, Italy; 3 Veneto Oncology Institute, IOV-IRCSS Pathology and Surgery Units, Padova, Italy

## Abstract

**Introduction:**

The spontaneous rupture of a parathyroid adenoma accompanied by extracapsular hemorrhage is a rare, potentially fatal, condition and is a cervicomediastinal surgical emergency.

**Case presentation:**

This report describes an atypical two-step spontaneous rupture of an asymptomatic parathyroid adenoma in a 56-year-old Caucasian woman who presented with a painful mass in the right side of her neck.

**Conclusion:**

Based on this case report and similar cases reported in the medical literature, a diagnosis of extracapsular parathyroid hemorrhage should be considered when a non-traumatic sudden neck swelling coexists with hypercalcemia and regional ecchymosis.

## Introduction

Hypercalcemia is the most common clinical sign of a parathyroid adenoma [1]. Hemorrhagic infarction may occur both in a parathyroid adenoma and in hyperplastic parathyroid glands, whereas extracapsular hemorrhage due to hyperplasia, adenoma, or cancer is an uncommon but threatening occurrence, resulting in a cervicomediastinal hematoma and is often associated with severe blood calcium imbalance. To date, 27 cases have been reported in the literature (usually as single cases or small case series) and none of them describe a two-step clinical picture of bleeding from the parathyroid gland (Table 1) [1]-[25].

**Table 1:** Clinicopathological features in case reports of extracapsular parathyroid hemorrhages

Author (year)	#	Clinical features	Ca^2+^ (normal values)	T	Histology (Ø = cm)
Capps R (1934) [2]	1	Weakness, sore throat, cervical swelling/ecchymosis, dysphagia, dyspnea	NA	5 weeks	Adenoma (Ø = 7)
Berry BE (1974) [3]	1	Weakness, retrosternal pain, signs of superior vena caval compression	NA	1 day	Adenoma (Ø = 5)
Santos GH (1975) [4]	1	Retrosternal pain, dizziness, hypotension, hypercalcemia	NA	1 day	Adenoma (Ø = 8)
Jordan FT (1981) [5]	1	Anterior cervical pain, swelling and ecchymosis, dysphagia	13.3 mg/dL	1 month	Hyperplasia (Ø = 6)
Roma J (1985) [6]	1	Hoarseness, dysphagia, cervical swelling, cervical-thoracic ecchymosis	3.00 mmol/L	1 day	Hyperplasia
Simcic KJ (1989) [7]	1	Painful cervical swelling, dysphagia, dyspnea	4 mmol/L (2.14-2.52)	2 weeks	Adenoma (Ø = 4.5)
Massard JL (1989) [8]	1	Difficulty swallowing, dysphonia, cervical pain, cervical ecchymosis	3.15 mmol/L	10 hours	Adenoma
Hotes LS (1989) [9]	1	Hoarseness, dysphagia, discomfort in the anterior area, ecchymosis	Normal	3 days	Adenoma
Alame A (1990) [10]	1	Difficulty swallowing, dysphonia, cervical-thoracic ecchymosis	2.86 mmol/L	1 day	Adenoma
Mantion G (1990) [11]	1	Dysphagia, dyspnea, ecchymosis	2.67 mmol/L	2 days	Adenoma (Ø = 2.5)
Amano Y (1993) [12]	1	Hoarseness, dysphagia, cervical swelling and ecchymosis	Normal	2 days	Adenoma (Ø = 5.6)
Korkis AM (1993) [13]	1	Hoarseness, dysphagia, cervical swelling and cervical-thoracic ecchymosis	11.6 mg/dL	1 day	Adenoma (Ø = 4)
Jougon J (1994) [14]	1	Dysphagia, cervical swelling and cervical-thoracic ecchymosis	2.9 mmol/L	1 day	Adenoma (Ø = 3)
Menegaux F (1997) [15]	1	Cervical pain, dysphagia, dyspnea	2.62 mmol/L	1 day	Adenoma
Hellier WPL (1997) [16]	1	Dysphagia, dysphasia, cervical-thoracic ecchymosis	Elevated	1 day	Adenoma
Ku P (1997) [17]	1	Hoarseness, cervical pain and ecchymosis	3.15 mg/dL	1 day	Adenoma (Ø = 2)
Kihara M (2001) [18]	1	Painful cervical swelling and cervical-thoracic ecchymosis	Normal	1 month	Adenoma (Ø = 2)
Kozlow (2001) [19]	1	Dysphagia, odynophagia, cervical swelling	11.3 mg/dL (8.4-10.2)	7 days	Adenoma
Nakajima J (2002) [20]	1	Retrosternal pain, cervical-thoracic ecchymosis	Normal	3 weeks	Adenoma (Ø = 3.5)
Govindaraj S (2003) [21]	1	Hyper-normo-calcemia, right-sided headaches, severe throat pain	13 mg/dL	2 weeks	Adenoma
Taniguchi I (2003) [22]	1	Cervical swelling, pain, dysphagia, cervical-thoracic ecchymosis	NA	1 month	Cyst (Ø = 6)
Maweja S (2003) [23]	2	Painful cervical swelling and cervical-thoracic ecchymosis	2.57; 2.80 mmol/L	2 days	Adenoma
Tonerini M (2004) [24]	1	Painful cervical swelling	NA	1 day	Adenoma
Akimoto T (2005) [25]	1	Left pleural effusion in IPT II at chest X-ray and computed tomography scan	Elevated	1 day	Hyperplasia
Devezè A (2006) [1]	2	1. Dysphagia cervical hematoma, hypercalcemia	2.57 mmol/L	1 week	1. Adenoma
		2. Latero-cervical pain and ecchymosis		5 days	2. Adenoma
Merant-Boschin I (2009)	1	Painful cervical swelling, dysphonia, dyspnea	3.18 mmol/L (2.10-2.55)	2 days	Adenoma (Ø = 4.0)

**Notes:** #, number of cases described; [Ca^2+^]: calcium level; T: time of onset; Normal: in the normal range; NA: Not assessed.

Patients usually present with a palpable lateral neck mass with signs of ecchymosis, appearing slowly 24 to 48 hours after the sudden onset of neck discomfort, dysphagia, dyspnea, or hoarseness [19,24]. Such an emergency requires immediate surgical treatment and the prognosis depends on the extent and location of the hematoma.

This case report describes a patient who experienced a two-step spontaneous rupture (with extracapsular bleeding) of a large (probably long-standing) asymptomatic parathyroid adenoma. To the best of our knowledge, this is the first report of such an atypical modality of parathyroid adenoma rupture.

## Case presentation

**Figure 1:**
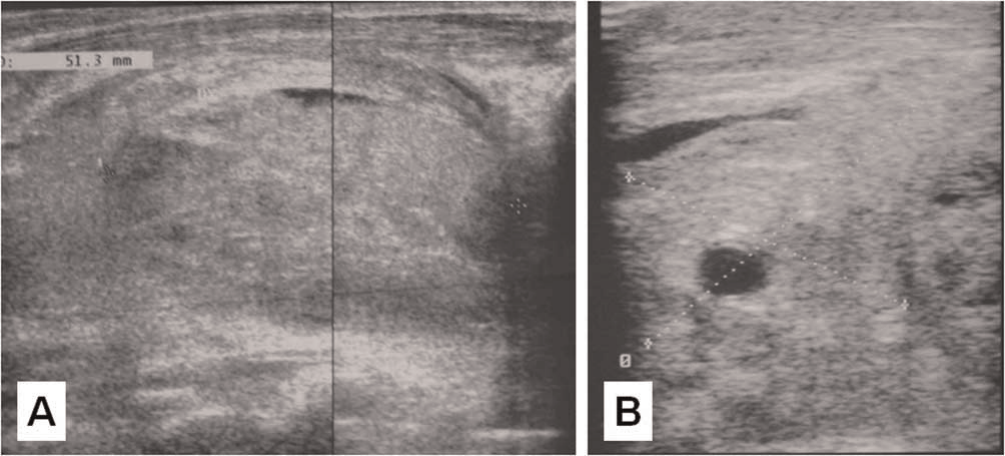
**Neck ultrasound on admission**. Longitudinal and transverse views demonstrating a 51.3 mm nodular iso-echoic lesion, of dyshomogeneous structure and hemorrhagic pattern (**A**). The lesion surrounds the right common carotid artery and internal jugular vein, and is located posterior to the right lobe of the thyroid, with ill-defined posterior margins (**B**).

In April 2007, a 56-year-old Caucasian woman with a painful, right-sided neck mass presented to a private practitioner. Ultrasound (US) suggested a clinical diagnosis of subacute thyroiditis, which was not supported by subsequent laboratory tests (C-reactive protein 1.9 mg/L; leukocytes 9700/μL; thyroid hormones within normal range; antithyroid auto-antibodies negative). Two days later, the patient had an exacerbation of the latero-cervical pain which prompted a repeat US of the neck, which revealed an iso-echoic lesion (51.3 mm in size), apparently included within an enlarged right thyroid lobe (83.5 mm). The lesion was interpreted as an intrathyroid hematoma (Figure 1A, B) and the opinion of a neck surgeon (MRP) was requested. The patient's medical history was collected at this time and included a severely diminished bone mass treated with bisphosphonate, though no information on bone metabolism was provided. History ruled out any regional traumatic event. The patient seemed quite anxious and dysphonetic but not dyspnoeic. Physical examination revealed a tender right-sided cervical mass, extending from the right mandibular arch to the thoracic inlet.

**Figure 2:**
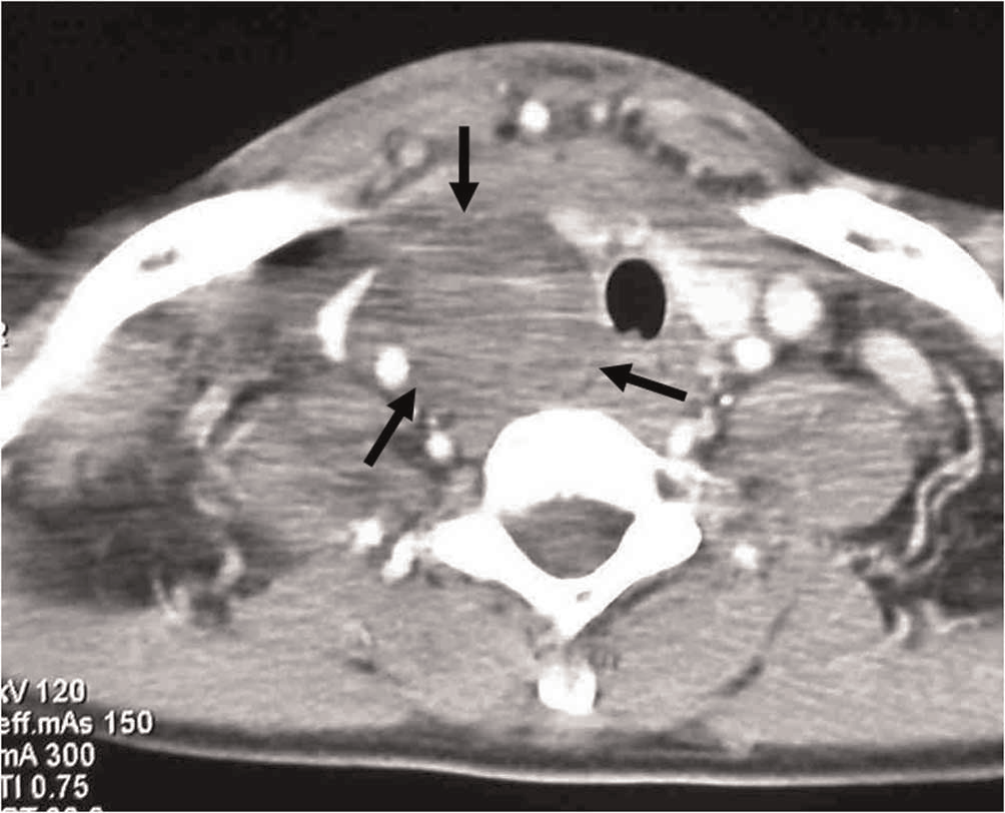
**Computed tomography scan on admission (at thoracic inlet level) showing signs of cervical-mediastinal hematoma (black arrows) in the right prevertebral and paratracheal space**. A marked midline shifting and compression of the trachea is evident.

**Figure 3:**
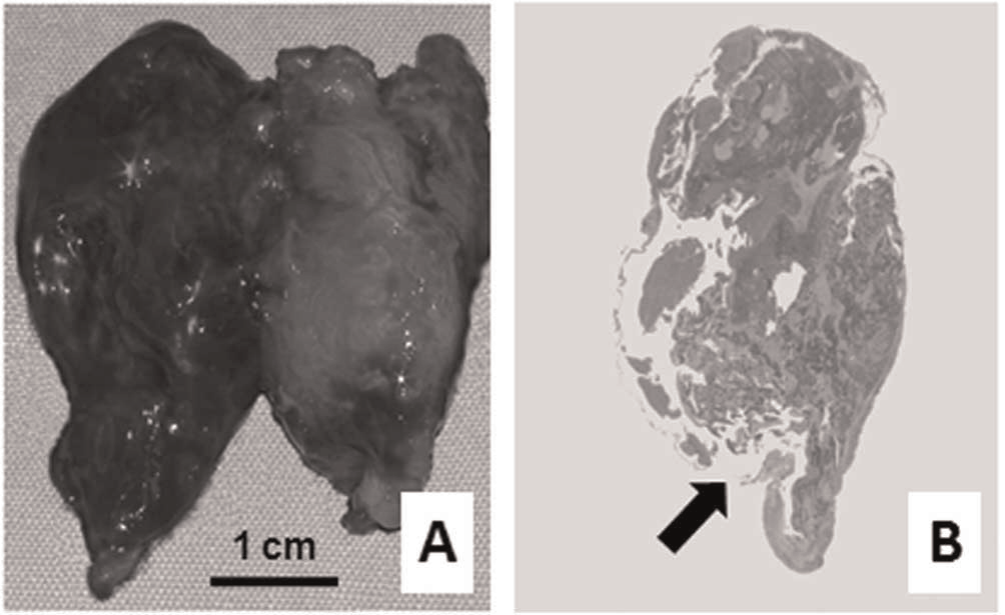
**Gross specimen consisting of red-brown parathyroid adenoma of the upper-right parathyroid gland (left) and right thyroid lobe (right) (**A**)**. Gross histological section showing the whole parathyroid gland and the rupture of its capsule (arrow) (**B**). Original magnification 2×.

The patient was referred to the Special Surgical Pathology Department at Padova University Hospital, where computed tomography (CT) showed a laterocervical hemorrhagic lesion, extending from the lateral neck to the right prevertebral/paratracheal spaces (Figure 2); a distinct midline shift and compression of both the hypopharynx and the trachea were also documented. During the CT procedure, the patient suffered from severe respiratory distress with dyspnea and she was immediately referred for surgical treatment, where an ovoid, hemorrhagic mass (4.0 × 2.4 × 1.3 cm, weight 8.1 g) was revealed posterior to the right thyroid lobe. Laboratory tests (conducted during the surgical procedure) demonstrated severe hypercalcemia (3.18 mmol/L; normal range: 2.10 to 2.55 mmol/L) with a decrease in hemoglobin level (12.0 g/dL). Surgery consisted of hematoma evacuation, parathyroidectomy and "en bloc" right thyroid lobectomy (Figure 3A). There was no evidence of regional lymph node involvement. The surgery was curative and both serum calcium and parathyroid hormone (PTH) levels quickly dropped to within the normal range (at discharge: calcium 2.29 mg/dL; PTH 52 pg/mL).

Gross section of the surgical specimen revealed a three-layered lesion consisting of peripheral areas of (partially fluid) hemorrhagic material, invading a more internal, compact (partially organized) zone around the core of the specimen, which consisted of necrotic parathyroid remnants (Figure 3B). Multiple gross samples were obtained for histological assessment, which showed an extensively hemorrhagic chief cell parathyroid adenoma surrounded by a loosely organized hemorrhagic and fibrous reaction, which became frankly hemorrhagic in the tissue samples obtained from the periphery of the resected mass.

A 9-month follow-up including clinical evaluation, serology and US, revealed no clinical abnormalities.

## Discussion

Spontaneous neck hemorrhage is a rare, frightening surgical emergency, usually resulting from the traumatic rupture of vessels or from generally spontaneous thyroid or parathyroid extraglandular bleeding. A parathyroid intra- and extracapsular hemorrhage is a serious, potentially fatal, complication of parathyroid gland enlargement due to hyperplasia, adenoma or cancer. The physiopathological mechanisms behind such non-traumatic bleeding are not known. They probably stem from an imbalance between cell growth and blood supply, a situation prone to the onset of necrotic-hemorrhagic foci, which may ultimately spread outside the glandular structure; this mechanism has been considered similar to the apoplexy seen in other endocrine neoplasia [26].

Capps first documented a fatal case of spontaneous massive parathyroid hemorrhage with cervical/mediastinal infarction in 1934, which was only assessed at post-mortem examination [2]. To date, 27 cases have been reported in the literature, with variable clinical presentations [1]-[25], the variability concerning the endocrine clinical syndrome at presentation (usually a hypoparathyroidism of sudden onset), the size of the hemorrhagic mass and the timing of the cervical/thoracic bleeding. According to Simcic *et al.,* however, a clinical triad consisting of acute neck swelling, hypercalcemia, and neck and/or chest ecchymosis strongly point to this clinical hypothesis [7]. Table 1 summarizes the cases reported in the literature as at December 2007. The table refers strictly to cases featuring extracapsular parathyroid bleeding, confirmed on histology and excluding cases relating to neck traumas. The group of cases considered shows a significant variability in both clinical presentation including symptoms, time of onset and laboratory data such as calcium levels.

The differential diagnosis of non-traumatic lateral-neck bleeding involves thyroid lesions, cyst or nodular goiter, subacute thyroiditis, and parathyroid conditions, such as adenoma, hyperplasia or cancer [9,14,24]. As in this patient, it may be difficult, if not impossible, to distinguish clinically between a thyroid and parathyroid origin of the problem, and even imaging techniques such as CT and US may be bewildering. In this respect, a primary parathyroid involvement should be considered when a clinical syndrome centered in the lateral area of theneck, such as cervical swelling, cervical-thoracic ecchymosis, dysphagia and dyspnea, coexists with blood calcium imbalance.

In our patient, the clinical history of a bland presentation, quiescent interval and final emergency, and the pathological features of the resected mass are both consistent with a spontaneous rupture of a parathyroid adenoma in two successive stages. As described in many other parenchymal organs here too we can assume that an initial episode of paucisymptomatic intracapsular bleeding progressed to a capsular rupture resulting in a massive cervical and/or mediastinal infarction.

## Conclusion

This case should alert physicians that parathyroid extracapsular hemorrhage needs to be considered among the list of non-traumatic surgical neck emergencies and, in line with the current literature, any neck swelling, variably associated with "mass" symptoms such as dysphagia and/or dyspnoea, in association with hypercalcemia and regional ecchymosis, should strongly point to this clinical hypothesis.

## Competing interests

The authors declare that they have no competing interests.

## Authors' contributions

All authors of this paper have participated directly in the planning, execution, or analysis of the study, and have read and approved the final version submitted.

## Consent

Written informed consent was obtained from the patient for publication of this case report and any accompanying images. A copy of the written consent is available for review by the Editor-in-Chief of this journal.

## References

[B1] Devezè A Sebag F Pili S Henry JF Parathyroid adenoma disclosed by a massive cervical hematoma Otolaryngol Head Neck Surg 2006 134 710 712 10.1016/j.otohns.2005.03.075 16564403

[B2] Capps R Multiple parathyroid tumors with massive mediastinal and subcutaneous hemorrhage: a case report Am J Med Sci 1934 188 800 805 10.1097/00000441-193412000-00007

[B3] Berry BE Carpenter PC Fulton RE Danielson GK Mediastinal hemorrhage from parathyroid adenoma simulating dissecting aneurysm Arch Surg 1974 108 740 741 4829792 10.1001/archsurg.1974.01350290102019

[B4] Santos GH Tseng CL Frater RW Ruptured intrathoracic parathyroid adenoma Chest 1975 68 844 846 10.1378/chest.68.6.844 1192872

[B5] Jordan FT Harness JK Thompson NW Spontaneous cervical hematoma: a rare manifestation of parathyroid adenoma Surgery 1981 89 697 700 7245031

[B6] Roma J Carrio J Pascual R Oliva JA Mallafre JM Montoliu J Spontaneous parathyroid hemorrhage in a hemodialysis patient Nephron 1985 39 66 67 10.1159/000183342 3969195

[B7] Simcic KJ McDermott MT Crawford GJ Marx WH Ownbey JL Kidd GS Massive extracapsular hemorrhage from a parathyroid cyst Arch Surg 1989 124 1347 1350 2684095 10.1001/archsurg.1989.01410110109023

[B8] Massard JL Peix JL Bizrane M Khalaf M Hugues B Cervico-mediastinal hemorrhage revealing parathyroid adenoma Presse Med 1989 18 1524 1525 2530517

[B9] Hotes LS Barzilay J Cloud LP Rolla AR Spontaneous hematoma of a parathyroid adenoma Am J Med Sci 1989 297 331 333 10.1097/00000441-198905000-00012 2655447

[B10] Alame A Solovei G Alame S Buyse N Glavier F Petit J Parathyroid adenoma revealed by extracapsular cervico-mediastinal hemorrhage Presse Med 1990 19 817 2140173

[B11] Mantion G Le Guillouzic Y Badet JM Gillet M Spontaneous cervical hematoma secondary to parathyroid adenoma Presse Med 1990 19 1197 2142291

[B12] Amano Y Fukuda I Mori I Kumoi T Hemorrhage from spontaneous rupture of a parathyroid adenoma: A case report Ear Nose Throat J 1993 72 794 799 8313863

[B13] Korkis AM Miskovitz PF Acute pharyngoesophageal dysphagia secondary to spontaneous hemorrhage of a parathyroid adenoma Dysphagia 1993 8 7 10 10.1007/BF01351471 8436023

[B14] Jougon J Zennaro O Acute cervico-mediastinal hematoma of parathyroid origin Ann Chir 1994 48 867 869 7702348

[B15] Menegaux F Boutin Z Chameau AM Dahman M Schmitt G Chigot JP Large cervical hematoma of parathyroid origin Presse Med 1997 26 1969 9536995

[B16] Hellier WP McCombe A Extracapsular haemorrhage from a parathyroid adenoma presenting as a massive cervical haematoma J Laryngol Otol 1997 111 585 587 10.1017/S0022215100137995 9231101

[B17] Ku P Scott P Kew J van Hasselt A Spontaneous retropharingeal haematoma in a parathyroid adenoma Aust N Z J Surg 1998 68 619 621 10.1111/j.1445-2197.1998.tb02117.x 9715147

[B18] Kihara M Yokomise H Yamauchi A Irie A Matsusaka K Miyauchi A Spontaneous rupture of a parathyroid adenoma presenting as a massive cervical hemorrhage: report of a case Surg Today 2001 31 222 224 10.1007/s005950170172 11318124

[B19] Kozlow W Demeure MJ Welniak LM Shaker JL Acute extracapsular parathyroid hemorrhage: case report and review of the literature Endocr Pract 2001 7 32 36 11250766 10.4158/EP.7.1.32

[B20] Nakajima J Takamoto S Tanaka M Takeuchi E Murakawa T Kitagawa H Fukayama M Parathyroid adenoma manifested by mediastinal hemorrhage: report of a case Surg Today 2002 32 809 811 10.1007/s005950200155 12203060

[B21] Govindaraj S Wasserman J Rezaee R Pearl A Bergman DA Wang BY Urken ML Parathyroid adenoma autoinfarction: A report of a case Head Neck 2003 25 695 699 10.1002/hed.10244 12884353

[B22] Taniguchi I Maeda T Morimoto K Miyasaka S Suda T Yamaga T Spontaneous retropharyngeal hematoma of a parathyroid cyst: report of a case Surg Today 2003 33 354 357 10.1007/s005950300080 12734730

[B23] Maweja S Sebag F Hubbard J Misso C Henry JF Spontaneous cervical haematoma due to extracapsular haemorrhage of a parathyroid adenoma: a report of 2 cases Ann Chir 2003 128 561 562 10.1016/S0003-3944(03)00184-6 14559311

[B24] Tonerini M Orsitto E Fratini L Tozzini A Chelli A Santi S Rossi M Cervical and mediastinal hematoma: presentation of an asymptomatic cervical parathyroid adenoma: case report and literature review Emerg Radiol 2004 10 213 215 10.1007/s10140-003-0317-0 15290495

[B25] Akimoto T Saito O Muto S Hasegawa T Nokubi M Numata A Ando Y Sohara Y Saito K Kusano E A case of thoracic hemorrhage due to ectopic parathyroid hyperplasia with chronic renal failure Am J Kidney Dis 2005 45 e109 114 10.1053/j.ajkd.2005.03.004 15957122

[B26] Howard J Follis RH Yendt ER Connor TB Hyperparathyroidism. Case report illustrating spontaneous remission due to necrosis of adenoma, and a study of the incidence of necrosis in parathyroid adenomas J Clin Endocrinol 1953 13 997 1008 10.1210/jcem-13-8-997 13069604

